# Management of presumed trematode-induced granulomatous intermediate uveitis

**DOI:** 10.1038/s41433-022-02336-4

**Published:** 2022-12-07

**Authors:** Rana Hussein Amin, Abdussalam Mohsen Abdullatif

**Affiliations:** Kasr Al-Aini Hospital, Cairo University, Giza, Egypt

**Keywords:** Uveal diseases, Parasitic infection

## Abstract

**Purpose:**

To describe the surgical management of presumed trematode-induced granulomatous intermediate uveitis (PTIGIU) not responding to medical treatment in controlling the inflammation.

**Methods:**

A prospective, interventional, single-center study in which patients with a history of fresh canal water contact and PTIGIU were enrolled. All patients underwent lensectomy–pars plana vitrectomy (PPV) and post-operative control of inflammation, functional and anatomical outcomes were assessed.

**Results:**

Fifteen eyes of 12 patients were included in the study with median age of 11.6 ± 4 yrs. Six months following lensectomy-PPV, inflammation was well controlled in all patients. The eyes were divided into two groups: Group A: 10 patients with an attached retina while Group B: 5 patients who were in the cicatricial stage with tractional retinal detachment. All patients in group A had CDVA of 20/40 or better, unlike patients in group B who failed to achieve a CDVA better than 20/70 throughout their follow-up. In group B, final anatomical success was achieved in only 40% with hypotony occurring in 20%.

**Conclusion:**

PTIGIU is associated with the presence of ciliary body granuloma which, if left untreated, can lead to drastic outcomes. Early lensectomy-PPV represents a viable management option in cases resistant to medical treatment, with a favorable outcome.

## Introduction

During the past two decades, parasitic infestation from swimming in fresh canal water has been well-recognized as an important cause of infectious uveitis in patients from developing countries [1–4]. The term “presumed” is usually used to describe such uveitic entity since fragments and genetic material of the parasites could be isolated from only some of the surgically removed granulomas without signs of systemic parasitic disease [3–6]. Presumed trematode-induced uveitis usually develops in adolescent children with different presentations, the most common of which is anterior uveitis with anterior chamber (AC) granuloma [1, 3, 4]. The other presentations reported include variable degrees of intraocular inflammation in association with subconjunctival [2], corneal, retrocorneal, and iris granulomas [4]. Although their incidence is not known and seems to be underestimated due to lack of reporting, yet such granulomas can also arise inside the ciliary body (CB) associated with intermediate uveitis (IU) or even in the form of posterior uveitis [3, 4].

Similar to ocular toxocariasis, presumed trematode-induced granulomatous intermediate uveitis (PTIGIU) presents with vitritis and complicated cataract (active stage) which, if left untreated, progresses to tractional retinal detachment (TRD) from the vitreoretinal traction (cicatricial stage) and eventually phthisis [7]. Accordingly, timely diagnosis of such patients during the active stage is essential for early proper management to avoid complications that might eventually result in blindness. Patients with trematode-induced AC granulomas are usually managed via various combinations of systemic antiparasitic treatment, peribulbar anterior subtenon steroids injections, and surgical granuloma excision [2, 3, 8, 9]. However, to date, there has been no published data regarding the management of patients with PTIGIU that do not respond to regional steroids and/or systemic treatment for a duration of 3 months.

The aim of our study was to describe the surgical management of PTIGIU not responding to medical treatment and to assess its efficacy in controlling inflammation and anatomical and functional outcomes.

## Methods

This is a prospective interventional study in which the patients with PTIGIU not responding to medical treatment attending the Ocular Immunology and Uveitis Clinic, Ophthalmology Department, Kasr Al-Aini Hospital, Cairo University between January 2021 to January 2022 were enrolled. All tenets of the Declaration of Helsinki were followed, and informed consent was obtained by the guardians of the patients or the patients when applicable. The study protocol was revised and approved by the Ophthalmology Department scientific committee, Cairo University.

### Inclusion criteria

For recruiting PTIGIU patients were based on presence of all of the following together: (1) history of fresh canal water contact in one of the branches of the River Nile prior to the onset of uveitis, (2) signs of IU with the detection of CB granuloma by ultrasound biomicroscopy (UBM), (3) absence of clinical signs or work-up suggestive of any other cause of granulomatous IU, and (4) failure of medical treatment. Failure of medical treatment was defined as persistent or recurrent anterior (flare and cells >+1) and/or posterior segment inflammation (>Grade I vitritis) following regional ocular steroids injections plus oral steroids for 8–12 weeks as reported in previous literature [8].

### Exclusion criteria

Patients with a known cause of granulomatous uveitis other than trematodal infection (such as toxoplasmosis, toxocariasis, sarcoidosis, tuberculosis), previous ocular surgery, ocular trauma, or follow-up <6 months.

### Baseline data

For all patients, we documented the age, gender, eye laterality, duration of disease, and the treatment received (topical, regional, and systemic). Careful review of systems and thorough investigations were done (to exclude other causes of granulomatous uveitis) in the form of complete blood picture, stool and urine analysis, fluorescent treponemal antibody absorbent test, angiotensin-converting enzyme, tuberculin skin testing, toxocara antibodies, toxoplasma antibodies, and chest X-ray. In cases where investigations yielded positive results for another possible cause of granulomatous disease, such as a positive tuberculin skin test or toxoplasma antibodies, the patients were excluded from the study.

Corrected distance visual acuity (CDVA), intraocular pressure measurement, and ophthalmic findings with slit-lamp examination were recorded including the AC and vitreous inflammatory grades according to the standardized uveitis nomenclature (SUN) classification [10]. Dilated fundus examination with slit-lamp biomicroscopy and indirect ophthalmoscopy were done to document the presence of retinal detachment, macula status, and grades of proliferative vitreoretinopathy (PVR). All patients underwent UBM and the presence of CB granuloma and its extent were recorded. Also, B scan ultrasonography (US) was done if media opacities precluded fundus examination.

### Surgical intervention

**Fig. 1:**
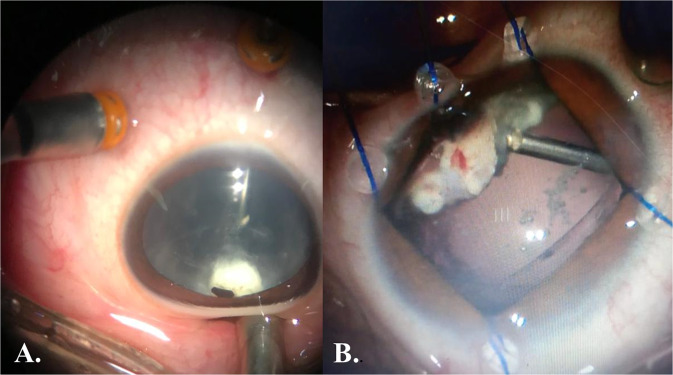
Intraoperative picture during the vitrectomy procedure. **A** With scleral indentation at the site of ciliary body granuloma showing the latter behind the partially cataractous lens. **B** During debulking of the ciliary body granuloma using the vitrectomy probe after pars plana lensectomy with preservation of capsular rim for secondary implantation. Iris hooks help improve visualization in poorly dilatable pupils.

All surgeries were done under general anesthesia. Any posterior synechiae were released and the pupil was dilated with the help of iris hooks whenever necessary. Lensectomy without IOL implantation was done in all patients through an anterior or posterior approach. Vitrectomy was performed using Constellation vitrectomy machine (Alcon Laboratories, Inc., Fort Worth, TX, USA) and a 23-gauge cannula system. Cannulas were inserted at sites away from the CB granuloma location pre-determined by UBM. Posterior capsulotomy and anterior vitrectomy were done followed by focusing the microscope on the CB granuloma with the help of deep peripheral scleral indentation corresponding to the site of the granuloma (Fig. 1A). Anterior and posterior capsules were sacrificed at the site of the granuloma followed by debulking of the latter as much as possible, taking care not to injure the CB as the granuloma is always amalgamated with it. Diathermy was applied to the remaining parts of the granuloma as well as any bleeding points. All patients were left aphakic with a plan for secondary implantation at a later date when the ocular inflammation subsided. Using an indirect wide-angle viewing system (BIOM, Oculus, Wetzlar, Germany), core vitrectomy was done followed by posterior hyaloid detachment aided with triamcinolone acetonide (TA) aqueous suspension (Kenacort 40 mg/ml, Bristol-Myers Squibb Company, Cairo, Egypt). For adherent posterior hyaloid, diamond-dusted tano scraper or serrated forceps were used. Posterior hyaloid was peeled up to the posterior border of vitreous base or up to the line where it was strongly adherent. Under scleral indentation, complete vitreous base shaving was meticulously performed especially at the site of the CB granuloma (Fig. 1B). If retina is attached, fluid air exchange was done and the eyes were left on air followed by suturing of the sclerotomies with vicryl 8/0 sutures.

If there was TRD, all of the epiretinal membranes were peeled off the surface of the retina. Failure of retinal flattening indicated the presence of intrinsic retinal shortening necessitating retinectomy. A relaxing circumferential retinectomy using vitreous cutter after cautery application was extended as needed to relieve retinal shortening. Retina anterior to the retinectomy site was removed to prevent neovascularization and re-proliferation. After complete retinal re-attachment was achieved, argon laser photocoagulation was applied 360-degrees, at the edges of the retinectomy and to any retinal breaks, followed by direct PFCL-silicone oil (SO) (1000 centistokes) (DORC, Zuidland, Netherland) exchange. Sclerotomies were sutured with vicryl 8/0 sutures, whenever needed.

All eyes received topical antibiotics with topical prednisolone acetate 1% eyedrops given every 2 hours for the first 48 hours followed by 5 times daily to be tapered according to AC reaction in addition to antibiotic–steroid ointment at bedtime.

### Post-operative follow-up data

During post-operative visits, a complete ophthalmological examination documenting any post-operative complications and the course of inflammation was done on the first post-operative day, weekly for 1 month, then monthly thereafter for 6 months. At 3 months post pars plana vitrectomy (PPV), UBM was done to follow-up the size of the CB granuloma. Secondary IOL implantation was scheduled 3 months following vitrectomy in cases of controlled inflammation. In silicone oil-filled globes with stable attached retinae, silicone oil removal with secondary IOL implantation was performed 3 months after the initial surgery.

### Main outcome measures

1. Degree of inflammation anteriorly and posteriorly.
2. Functional outcome: CDVA measured using a Snellen chart.
3. Anatomical outcome: rates of anatomical success defined as stable, complete retinal re-attachment without internal tamponade at 3 months post-vitrectomy.

### Statistical methods

Statistical analysis was done using SPSS computer software package, version 15.0, 2006, Echosoft Corporation, USA. Quantitative data were expressed as mean ± SD or median ± interquartile range (IQR) and differences between groups were assessed Mann–Whitney U test. While qualitative data were expressed as frequencies and percentages and chi-square was used as a test of significance. All tests were two-tailed and considered significant at *p* < 0.05.

## Results

**Fig. 2:**
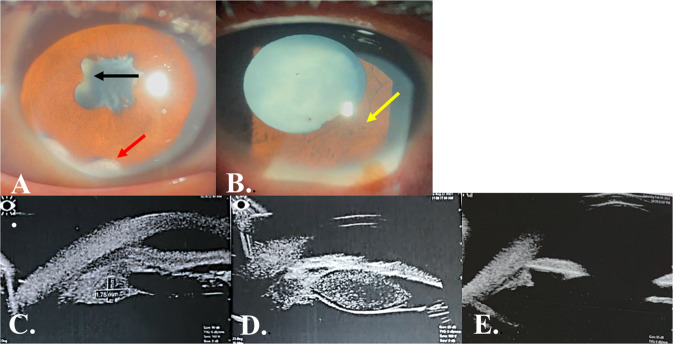
Anterior segment photo and ultrasound biomicroscopy revealing. Anterior segment photo showing **A** Temporal localized cataract (black arrow) corresponding to the site of ciliary body granuloma in a patient with previously healed anterior chamber granuloma (red arrow). **B** Total lens opacity with localized forward iris bulge (yellow arrow) corresponding to the site of ciliary body granuloma. Ultrasound biomicroscopy revealing **C** homogenous lesion with medium-reflectivity (ciliary body granuloma) restricted to ciliary body measuring ~1.78 mm thick, **D** ciliary body granuloma extending in front of the lens and pushing the iris forwards, **E** healed ciliary body granuloma, 3 months following PPV.

Fifteen eyes of 12 patients with PTIGIU were included in the study with three patients having bilateral disease. All patients had a history of recent canal water contact prior to the onset of uveitis with a negative review of systems and normal results of all systemic investigations. The mean age was 11.6 ± 4 years with most of the patients being in the adolescent ages (range 8–17 years) with 83% of the patients were males. The median pre-operative CDVA was 0.03 and the duration of uveitis prior to presentation was 5 months (range 2–24 months). All patients except one had cataracts whether localized (Fig. 2A) at the site of granuloma or in the form of total lens opacity (Fig. 2B). Demographics and baseline ocular features are shown in Table 1.

**Table 1:** Patients’ characteristics and clinical features at presentation.

Features	
Age (yrs); mean (SD)	11.6 ± 4
Gender; *n* (%)	
Female	2 (17)
Male	10 (83)
Duration of the disease before vitrectomy (months); median (range)	5 (2–24)
Treatments received before surgical intervention	
Systemic steroids (Prednisolone 1 mg/kg/day)	12 (100)
Antiparasitic drugs	1 (6.7)
Peribulbar steroids (Subtenon Triamcinolone Acetonide 4 mg/ml)	8 (53.3)
Topical steroids (Prednisolone acetate 1% eyedrops) and cycloplegics	12 (100)
CDVA; median (range)	20/630 (20/20000–20/125)
IOP (mmHg); mean (SD)	11.3 ± 3
Anterior chamber cells grading median (range)	2 (1–3)
Posterior synechiae, *n* (%)	13 (86.6)
Lens status, *n* (%)	
Clear	1 (6.67)
Localized cataract	7 (46.7)
Total lens opacity	7 (46.7)
Vitritis grading, *n* (%)	
0	0
I	0
II	0
III	4 (26.7)
IV	4 (26.7)
Cannot be assessed clinically	7 (46.6)

*CDVA* corrected distance visual acuity, *IOP* intraocular pressure.

In cases of total lens opacification precluding fundus viewing, ocular US revealed dense vitritis and additional tractional retinal detachment in 33.3%. Pre-operative UBM showed well-circumscribed, homogeneous medium-reflectivity mass (granuloma) localized for one to two clock hours located in the outer half of the CB in all cases (Fig. 2C, D).

After failure of medical treatment (summarized in Table 1), all cases were operated by a single surgeon (A.M.A). During the vitrectomy, the CB granulomas were visualized after pupillary dilation and cataract removal as whitish elevated masses at the pars plana.

At the first month postoperatively, median anterior chamber (AC) and posterior chamber reaction of +0.5 cells were observed that resolved completely by the second month onwards with a clear vitreous cavity without recurrence of inflammation throughout the follow-up period. One eye (6.7%) showed recurrent inflammation at the 3rd month. UBM was done at 3 months and showed healed CB granuloma with no evidence of any inflammation in both groups (Fig. 2E).

The patients were divided into two groups: Group A which included 10 patients (66.7%) with an attached retina or small localized TRD at the ora serrata, while Group B included 5 patients (33.3%) who were in the cicatricial stage with inferior TRD. The duration of uveitis prior to presentation was significantly different between the two groups with later presentation in group B (group A median = 3 months, range 3–6 months; group B median = 24 months, range 9–24 months, *p* = 0.003). The median pre-operative CDVA was significantly better in group A compared to group B (0.005 and 0.001 for group A and B, respectively, *p* = 0.018). Cataract density was higher in Group B with higher percentage of total lens opacity (group A = 28.5%, group B = 40%, *p* = 0.5).

**Fig. 3:**
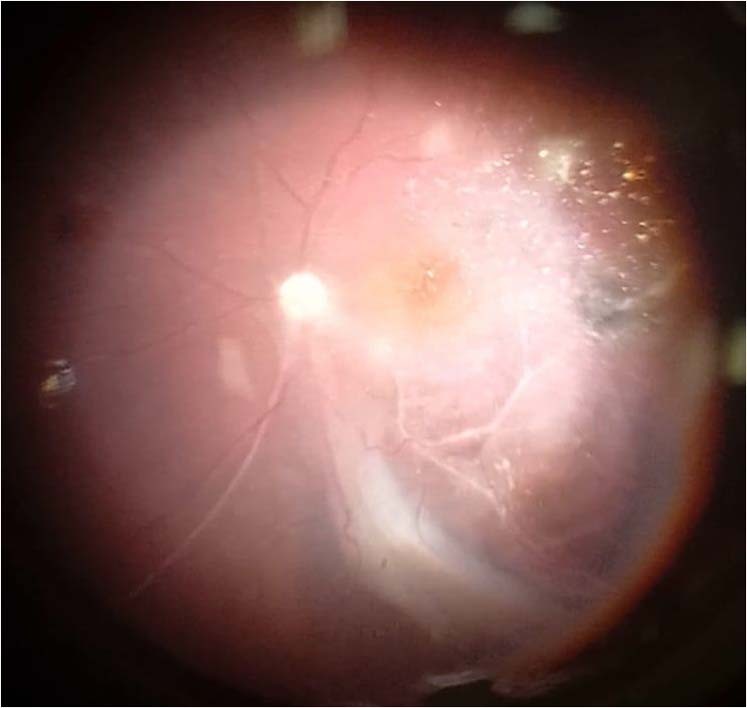
Intraoperative fundus photo of a patient in Group B showing tractional retinal detachment, inferior retinal fold, and subretinal bands extending to the optic disc.

In group A, surgeries were completed without complications and left on air as a tamponade in all cases. In group B, four eyes had macula-off retinal detachment (80%). The median number of detached quadrants was 2 and PVR > C with intrinsic retinal shortening was found in all eyes that required a relaxing circumferential retinectomy to reattach the retina. Subretinal bands extending to the optic disc with peripheral granulomas were found in two eyes (40%) (Fig. 3). One of these two patients had inferior macular dragging.

Regarding the visual results, the vision improved significantly in group A (CDVA 0.6, 0.75, and 0.75 at 1, 3, and 6 months, respectively, *p* values <0.05), while in group B, vision showed nonstatistical improvement (CDVA 0.01, 0.02, and 0.06 at 1, 3, and 6 months respectively, *p* values >0.05). Accordingly, there was a statistically significant difference between the two groups in the CDVA at all visits (*p* values <0.05). At the last recorded follow-up visit (6th month postoperatively), all patients in group A had CDVA of 20/40 or better, unlike patients in group B who failed to achieve a CDVA better than 20/70 throughout their follow-up (Table 2).

**Table 2:** Visual acuity at the final visit.

	Group A (*n* = 10)	Group B (*n* = 5)	*p* value
Final visit CDVA, median (range)	20/32 (20/40-20/25)	20/400 (20/2000 – 20/80)	0.002
No. of eyes (%)			
>20/70	10 (100)	0 (0)	0.00
20/400 to 20/70	0 (0)	2 (40)	0.04
<20/400	0 (0)	3 (60)	0.009

*CDVA* corrected distance visual acuity.

In group A, retina remained stable for the whole follow-up period while in group B, after the primary surgery, 60% (three eyes) showed recurrence as a result of PVR-related RRD and underwent a second vitrectomy with silicone oil tamponade. The median interval between the primary vitrectomy and PVR-related RRD was 4 months. Median number of procedures to achieve complete retinal attachment was 2. Silicone oil was removed at 3 months and final anatomical success was achieved in 40% (two eyes). Hypotony occurred in 20% (one eye) and globe survival was achieved in 80% (four eyes).

Regarding post-operative complications in group A, one patient had steroid-induced ocular hypertension (10%) controlled on topical anti-glaucoma drops while another patient had an epiretinal membrane (10%). In group B, ocular hypertension developed in 40% (two eyes) that was controlled medically, macular dragging in 20% (one eye) and band keratopathy in 20% (one eye).

## Discussion

Trematode-induced uveitis is a well-recognized cause of intraocular inflammation in children with a history of swimming in tributaries of the River Nile in rural villages [3, 4, 8, 9]. Our case series presented a less common presentation than the notorious AC granuloma in which the granuloma was located in the CB instead of the AC. It is still unknown why the granuloma developed particularly at this site, yet perhaps the rich vascularity of the CB is an important contributing factor considering that the larval/egg antigens are probably delivered to the eye via the uveoscleral blood vessels [11, 12]. The inflammatory reaction induced by these antigens attracts inflammatory cells [1, 3] giving rise to a mass-like lesion that can be visualized and accurately localized by the UBM [12].

Histopathological analysis of ocular samples from patients who had undergone surgery revealed non-specific inflammation with neutrophils and collections of epitheliod cells, histiocytes, eosinophils and lymphocytes [1, 3]. Trematode teguments can sometimes be found in close association with the granulomas, yet not in all samples, possibly due to rapid disintegration [13]. The most certain objective way for diagnosing trematode-induced uveitis is by using molecular diagnostics to recover the pathogen’s DNA from ocular samples using PCR testing and custom-designed primers targeting specific trematode DNA [3, 14]. Unfortunately, even conventional PCR testing performed by Amin RM et al yielded positive results in only 42.8% of the cases [3]. Therefore, diagnosis of trematode-induced uveitis in most cases is presumed and mainly based on history of canal water contact, characteristic clinical patterns and exclusion of other causes of granulomatous uveitis [14].

The PTIGIU patients present with vitritis (active stage) then the cicatricial stage follows with TRD. The same way the AC granuloma is known to cause peripheral anterior synechiae with the formation of retrocorneal vascularized fibrous membrane [1, 4], the CB granuloma also excites a similar posterior, intense immune response with fibrous components resulting in various grades of fibrous tissue proliferation. Extension can occur anteriorly to the iris root and the lens as well as posteriorly to the periphery of the choroid and retina [4, 8, 15]. The longer the periods of untreated inflammation (group B patients), the worse the visual acuity of such patients owing to total lens opacification and the contraction of the fibrous elements responsible for the formation of cyclitic membranes, epiretinal proliferations, or subretinal fibrotic bands predisposing to TRD and eventually phthisis [1, 4]. Highly similar findings have been reported by Chen Q et al in their work on ocular toxocariasis in which patients had peripheral granulomas in the CB, peripheral retinal detachments, and tractional cyclodialysis [16]. Due to the similarities in clinical presentations of both parasites, negative serological testing for toxocara antibodies is usually helpful in differentiating between presumed ocular helminthic infection and ocular toxocariasis.

In uveitic CB granulomas, UBM has proven to be a valuable diagnostic tool to determine the exact location and size of the lesions (number of clock hours) in addition to any associated findings such as traction on the iris root, cyclodialysis, CB effusion and cyclitic membranes [17, 18]. In a study performed on ocular toxocariasis, UBM identified 95% of CB and peripheral vitreoretinal granulomas that were seen during vitrectomy [19]. El Hefny E et al recently performed UBM on presumed trematode-induced uveitis patients and demonstrated the presence of the 1 to 2 clock hours CB granuloma in 90% of their patients as a homogenous medium hyperreflective lesion that was commonly found inferiorly between 5 and 7 o’clock. Other findings that were noted included various degrees of anterior extension into the iris, atrophy of the ciliary processes and cyclitic membranes [15].

Treatment of presumed trematode-induced uveitis is challenging. The objective is to reduce inflammation and control the active stage in order to prevent the development of complications associated with the cicatricial stage. Therefore, prompt treatment is crucial for a favorable outcome. Current treatment modalities used include the following: (1) corticosteroids either topical, regional, or systemic; [8] (2) antimicrobial medications with controversial results; [9] (3) laser photocoagulation; [20] (4) surgical treatment for cases not responding to conservative treatment [1, 8, 9].

One of the proposed effective treatment methods for AC granuloma was the AC wash and surgical excision of the granuloma, especially in cases resistant to medical therapy [8, 9]. We applied the same rationale to our patients presenting with posterior granulomas at the CB by debulking the granuloma during PPV in order to reduce the inflammation and halt the progression of the fibrous reaction caused by the granuloma. Intraoperatively, it was impossible to remove all of the granuloma without causing significant damage to the CB and/or the retina. In cases of ocular toxocariasis, Zhang et al. [21] left the granuloma in situ during vitrectomy for fear of retinal damage, and the inflammation recurred in only 12.5% of their cases during the follow-up period. In our work, no recurrent inflammation was noted with resolution of anterior and posterior uveitic activities except for one eye and they remained quiescent during the entire follow-up period.

The role of PPV in the management of IU has been reported by many authors before whether as a diagnostic or a therapeutic procedure and was associated with a decrease in the severity of inflammation, recurrence rate and the need for immunosuppressives postoperatively [22–24]. The surgery is commonly performed as a therapeutic approach in uveitic cases complicated by epimacular membrane, TRD, rhegmatogenous retinal detachment and vitreous hemorrhage [21]. Yet, even in the absence of these complications, PPV was also found to be beneficial in improving visual activity and decreasing inflammation in cases of persistent vitreous opacities despite full systemic medical therapy [22, 23]. In cases of non-infectious IU, it has been suggested that vitrectomy may be effective in improving vitreous inflammation by removing the antigenic load and reducing the levels of cytokines and interleukins that activate the inflammatory cascade [23–25]. Accordingly, well-timed PPV after the failure of medical treatment had a beneficial effect on the course, and the prognosis of chronic uveitis and early surgery correlated with the improvement in VA [22–25].

The same result was reported in cases with infectious etiology such as ocular toxocariasis [21, 26–28] and toxoplasmosis [29] where PPV was performed as a therapeutic option to improve visual acuity. It seems that debulking of infectious granulomas with the removal of the vitreous gel helps in controlling the inflammation without the need for systemic medical treatment postoperatively [21, 26–28] which was similar to our results. The success of PPV in cases with toxocara IU was largely influenced by the pre-operative status of the eye. Ocular toxocariasis patients with poor pre-operative visual acuity, more aggressive inflammation, pre-operative TRD and traction macular involvement had worse visual prognosis compared to other patients with better visual acuity and attached retinae pre-operatively [21, 26]. These results are similar to the outcomes of our work in which group A had more superior anatomical and functional outcomes compared to the more advanced group B. The higher grades of PVR discovered intraoperatively in group B candidates were associated with higher rates of retinectomies, the need for silicone oil tamponade, recurrence of retinal detachment and post-operative complications. We believe that group B represents the more advanced, delayed, chronic untreated form of group A patients.

Therefore, early recognition of PTIGIU with early surgical management, prior to the development of fibrous bands that induce TRD, might be recommended as an effective treatment modality in these candidates. The longer the duration between the onset of symptoms and the presentation to the ophthalmologist, the more likely it is to find extensive fibrous reaction with anterior and posterior advancements and a poorer prognosis.

The limitation of this study is that it has a small sample of patients owing to the uncommon presentation of the disease. In light of our results, we suggest considering surgery as a primary treatment option in some patients with PTIGIU, and multicentre studies on a larger scale are needed to provide more robust evidence on the appropriate management of those young patients.

## Conclusion

Presumed trematode-induced uveitis can present with IU secondary to the inflammation caused by a granuloma in the CB. If left untreated, drastic complications can ensue. PPV represents one of the treatment modalities for trematode-induced uveitis in cases not responding to medical treatment. Better outcomes are achieved when the surgery is performed early in the disease course before TRD or cyclitic membranes develop.

### Summary

#### What was known before

- presumed trematode-induced granulomatous IU is associated with the presence of CB granuloma which, if left untreated, can lead to drastic outcomes.

#### What this study adds

- Early lensectomy-PPV represents a viable management option in cases resistant to medical treatment, with a favorable outcome.
